# Aryl Hydrocarbon Receptor Deficiency Enhances Insulin Sensitivity and Reduces PPAR-α Pathway Activity in Mice

**DOI:** 10.1289/ehp.1103593

**Published:** 2011-08-17

**Authors:** Chun Wang, Can-Xin Xu, Stacey L. Krager, Kathleen M. Bottum, Duan-Fang Liao, Shelley A. Tischkau

**Affiliations:** 1 Department of Pharmacology, Southern Illinois University School of Medicine, Springfield, Illinois, USA; 2 Institute of Pharmacy and Pharmacology, College of Pharmacy and Life Science, University of South China, Hengyang, Hunan, China; 3 Department of Internal Medicine, Southern Illinois University School of Medicine, Springfield, Illinois, USA; 4 Division of Stem Cell Regulation and Application, State Key Laboratory of Chinese Medicine Powder and Medicine Innovation in Hunan (Incubation), Hunan University of Chinese Medicine, Changsha, Hunan, China

**Keywords:** aryl hydrocarbon receptor, BMAL1, circadian rhythm, diabetes, dioxins, PPAR-α

## Abstract

Background: Numerous man-made pollutants activate the aryl hydrocarbon receptor (AhR) and are risk factors for type 2 diabetes. AhR signaling also affects molecular clock genes to influence glucose metabolism.

Objective: We investigated mechanisms by which AhR activation affects glucose metabolism.

Methods: Glucose tolerance, insulin resistance, and expression of peroxisome proliferator–activated receptor-α (PPAR-α) and genes affecting glucose metabolism or fatty acid oxidation and clock gene rhythms were investigated in wild-type (WT) and AhR-deficient [knockout (KO)] mice. AhR agonists and small interfering RNA (siRNA) were used to examine the effect of AhR on PPAR-α expression and glycolysis in the liver cell line Hepa-1c1c7 (c7) and its c12 and c4 derivatives. Brain, muscle ARNT-like protein 1 (*Bmal1*) siRNA and *Ahr* or *Bmal1* expression plasmids were used to analyze the effect of BMAL1 on PPAR-α expression in c7 cells.

Results: KO mice displayed enhanced insulin sensitivity and improved glucose tolerance, accompanied by decreased PPAR-α and key gluconeogenic and fatty acid oxidation enzymes. AhR agonists increased PPAR-α expression in c7 cells. Both *Ahr* and *Bmal1* siRNA reduced PPAR-α and metabolism genes. Moreover, rhythms of BMAL1 and blood glucose were altered in KO mice.

Conclusions: These results indicate a link between AhR signaling, circadian rhythms, and glucose metabolism. Furthermore, hepatic activation of the PPAR-α pathway provides a mechanism underlying AhR-mediated insulin resistance.

The World Health Organization (WHO 2011) has estimated that > 300 million people worldwide suffer from type 2 diabetes; epidemiological studies identify exposure to environmental toxicants as an independent risk factor for its development (Fujiyoshi et al. 2006; Kanagawa et al. 2008). Many structurally diverse compounds function through activation of the aryl hydrocarbon receptor (AhR) to contribute to the development of diseases (Poland et al. 1985; Safe 1986; Safe et al. 1985; Sato et al. 2008; Schmidt and Bradfield 1996; Swanson 2002; Van den Berg et al. 2006). The mechanisms by which long-term AhR activation causes diabetes are currently unknown.

As a member of the PER-ARNT-SIM (PAS) domain family of transcriptional regulators, ligand-activated AhR translocates to the nucleus and heterodimerizes with AhR nuclear translocator (ARNT), leading to toxic responses (Bock 1994). This well-described signaling pathway does not account for all effects of environmental toxicants, however. Crosstalk between AhR/ARNT and other nuclear receptors also contributes to toxicant-induced diseases (Puga et al. 2009; Shimba and Watabe 2009).

AhR/ARNT and the clock proteins (circadian locomotor output cycles kaput; CLOCK) and BMAL1 [brain, muscle ARNT-like protein 1; also called Arntl (aryl hydrocarbon receptor nuclear translocator-like)] share structural similarities and exhibit diurnal changes in multiple tissues (Richardson et al. 1998). AhR activation alters circadian rhythmicity and clock gene expression (Garrett and Gasiewicz 2006). Specifically, AhR activation disrupts CLOCK/BMAL1 activity and suppresses Period1 (Per1; also called period homolog 1) expression through interactions with BMAL1 (Xu et al. 2010). Chronic AhR activation also inhibits the responsiveness of the circadian clock to changes in environmental lighting (Mukai et al. 2008). Conversely, disruption of Per1 and Per2 expression alters the AhR signaling pathway (Qu et al. 2007, 2010). Collectively, the data reveal a complex relationship between AhR signaling and clock genes.

The circadian clock is also linked to metabolism. Key metabolic proteins, such as peroxisome proliferator–activated receptor-α (PPAR-α), exhibit circadian variation. Disruption of CLOCK and BMAL1 alters glucose tolerance and regulation of key metabolism genes (Marcheva et al. 2010; Oishi et al. 2005; Turek et al. 2005). More important, circadian regulation of BMAL1 and PPAR-α is interdependent (Canaple et al. 2006). Thus, PPAR-α may represent a critical link between the circadian clock and metabolism. The effects of PPAR-α on glucose metabolism are, however, equivocal. The PPAR-α agonist fenofibrate increases insulin sensitivity and decreases glucose (Haluzik et al. 2006). In contrast, recent studies strongly associate PPAR-α signaling with insulin resistance (Bernal-Mizrachi et al. 2003; Finck and Kelly 2002).

The aggregate literature compellingly implicates PPAR-α signaling, AhR activation, and circadian clock dysfunction in the development of type 2 diabetes in humans after exposure to environmental toxicants. AhR activation affects circadian rhythmicity and expression of clock genes, which regulate PPAR-α. In the present study we investigated the involvement of BMAL1 and PPAR-α in glucose metabolism after manipulation of AhR signaling using small interfering RNA (siRNA) or AhR agonists, as well as in AhR-deficient [knockout (KO)] mice. KO mice display increased responsiveness to insulin, decreased PPAR-α expression, and altered circadian rhythm of liver genes controlling glucose and fatty acid metabolism. The growing pandemic of type 2 diabetes, in part due to exposure to environmental toxicants, remains a major challenge in human health. This study provides important insight into mechanisms by which toxicants, acting through AhR, produce type 2 diabetes.

## Materials and Methods

*Animals.* Animals were treated humanely, with minimal suffering; animal protocols were approved by the institutional animal care committee of the Southern Illinois University School of Medicine. Male and female mice 4–24 weeks of age were used. Wild-type (WT; C57Bl/6J) and KO (Bradfield strain; Schmidt et al. 1996) littermates, obtained originally from R. Petersen (University of Wisconsin, Madison, WI, USA), were bred in our colony. Animals entrained to 12/12-hr light/dark cycles were decapitated, and livers were collected at 4-hr intervals starting at zeitgeber time (ZT) 0, the time of lights on, or ZT12, the time of lights off. Tissues were snap-frozen in liquid nitrogen and stored at –80°C until use.

*Cell culture and siRNA treatment.* The mouse Hepa-1c1c7 (c7), c12, and c4 liver cell lines (ATCC, Manassas, VA, USA) were cultured in DMEM (Dulbecco’s modified Eagle’s medium) Reduced Serum (HyClone, Logan, UT, USA) with 7.5% bovine growth serum (HyClone) and penicillin/streptomycin/amphotericin (MP Biomedicals, Solon, OH, USA) at 37°C in a humidified, 5% CO_2_ atmosphere. All culture experiments were repeated at least three times, with doses of drugs as indicated in the figures. Cultures were treated with β-napthoflavone (BNF; Sigma Chemical Co., St. Louis, MO, USA), 2,3,7,8-tetrachlorodibenzo-*p*-dioxin (TCDD) (L. Hansen, University of Illinois at Urbana-Champaign, Urbana, IL, USA), GW6471 (Sigma), and/or α-napthoflavone (ANF; Sigma). DMSO was the vehicle for all controls. Negative, *Ahr*, and *Bmal1* siRNAs [50 nM; Invitrogen, Carlsbad, CA, USA; for sequences, see Supplemental Material, Table, (http://dx.doi.org/10.1289/ehp.1103593)] were delivered into cells (4.0 × 10^4^) using Lipofectamine 2000 (Invitrogen) as described previously (Wang et al. 2009).

*Plasmid transfection.* Mouse *Bmal1* cloned into pCS2+MTK vector (from P. Sassone-Corsi, University of California–Irvine, Irvine, CA, USA) and human *Ahr* cloned in pcDNA3 vector (from G. Perdew, Pennsylvania State University, University Park, PA, USA) were transfected with or without siRNA into c7 cells using Lipofectamine 2000 and cultured for 48 hr.

*RNA extraction and analysis.* Total RNA was extracted from liver using TRI-Reagent (Sigma) according to the manufacturer’s protocol. After cDNA synthesis using Oligo(dT)15 Primer (Promega protocol; Promega, Madison, WI, USA), 5 μL cDNA (1:5 or no dilution for *Ppara* in cell experiments) was used for quantitative polymerase chain reaction (qPCR) using SYBR green (Quanta Biosciences, Gaithersburg, MD, USA) in a Smart Cycler rapid thermal cycler (Cepheid, Sunnyvale, CA, USA). Each assay included a no-reverse-transcriptase negative control. Primer sequences are given in the Supplemental Material, Table (http://dx.doi.org/10.1289/ehp.1103593). Relative standard curves were created for each gene primer as previously described (Xu et al. 2010); β-*actin* was used for normalization.

*Protein extraction and analysis.* Cell lines and liver tissue were harvested and homogenized in lysis buffer; total protein (80–100 μg) was separated using SDS-PAGE. Membranes were immunoblotted with antibodies to PPAR-α (1:1,000; Sigma) and AhR (1:1,000; Enzo Life Sciences, Farmingdale, NY, USA).

*Glucose tolerance test (GTT) and insulin tolerance test (ITT).* Studies were performed as described previously (Finck et al. 2005). The GTT and ITT were separated by at least 1 week. For the GTT, mice were injected with a 10% solution of d-glucose (1 g/kg body weight) after an overnight fast. For the ITT, mice received an intraperitoneal injection of porcine insulin (0.75 U/kg; Sigma) after a 6-hr fast. Tail blood was assayed using a TRUEtrack blood glucose meter (Nipro Diagnostics Inc., Fort Lauderdale, FL, USA).

*Lactate concentration measurements.* Cell medium and cell lysate were collected; lactate concentrations were measured using the L-Lactate Assay Kit for a 96-well plate (Eton Bioscience Inc., San Diego, CA, USA) according to the manufacturer’s instructions.

*Statistics.* We used Student’s *t*-test and analysis of variance for statistical analysis; *p* < 0.05 was considered statistically significant. All data represent mean ± SE.

## Results

**Figure 1:**
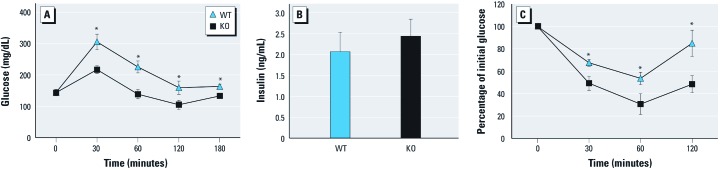
Glucose tolerance and insulin sensitivity in WT and KO mice. (*A*) GTT results for WT (*n* = 7) and KO (*n* = 4) mice. (*B*) Insulin levels in serum of WT (*n* = 4) and KO (*n* = 4) mice. (*C*) ITT results for WT (*n* = 8) and KO (*n* = 5) mice. Data are mean ± SE. *p < 0.05 compared with WT mice.

*Insulin sensitivity in* Ahr*-null mice.* We recorded weights of WT and KO mice monthly and found no effect of genotype on weight [see Supplemental Material, Figure 1 (http://dx.doi.org/10.1289/ehp.1103593)]. Fasting glucose levels were the same in WT and KO mice (Figure 1A). However, during GTT, glucose levels remained lower in KO mice for up to 180 min (Figure 1A). Serum insulin levels showed no significant difference between WT and KO mice at 20 min after glucose challenge (Figure 1B), suggesting that insulin release is similar in the two mouse strains. However, KO mice exhibited a larger reduction in glucose than did WT mice for up to 120 min after insulin challenge (Figure 1C), indicating enhanced insulin sensitivity in the KO mice.

**Figure 2:**
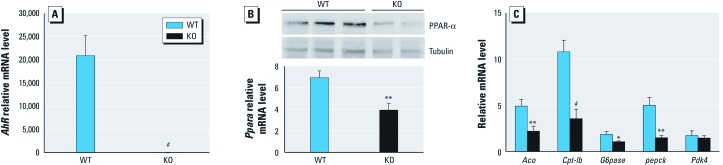
Expression of *Ahr*, *Ppara, *and metabolism genes in WT and KO mice. (*A*) qPCR analysis of *Ahr* mRNA. (*B*) PPAR-α protein (top; representative immunoblot from three WT and two KO animals) and *Ppara* mRNA (bottom) in WT and KO mice. (*C*) qPCR analysis of hepatic β-oxidation and gluconeogenic genes. All samples are from liver of WT (*n* = 8) and KO (*n* = 10) mice, collected at ZT8. Data are mean ± SE. *p < 0.05, **p < 0.01, and ^#^*p* < 0.001 compared with WT mice.

*Expression of key metabolic genes in KO and WT mice. Ahr* mRNA was essentially undetectable in KO mice (Figure 2A). *Ppara* mRNA and PPAR-α protein, which regulates fatty acid and glucose metabolism, were reduced in KO mouse liver (Figure 2B). Transcripts of the PPAR-α target genes acyl-CoA oxidase (*Aco*) and carnitine palmitoyl transferase 1b (*Cpt1b*) were decreased by 50% and 70%, respectively, in KO mice (Figure 2C). Transcripts for phosphoenolpyruvate carboxykinase (*Pepck*) and glucose 6-phosphatase (*G6pase*), which encode gluconeogenic enzymes, were also significantly reduced in the liver of KO mice (Figure 2C). In contrast, pyruvate dehydrogenase kinase, isozyme 4 (*Pdk4*) transcript, which encodes an inhibitor of glucose oxidation, did not change (Figure 2C).

**Figure 3:**
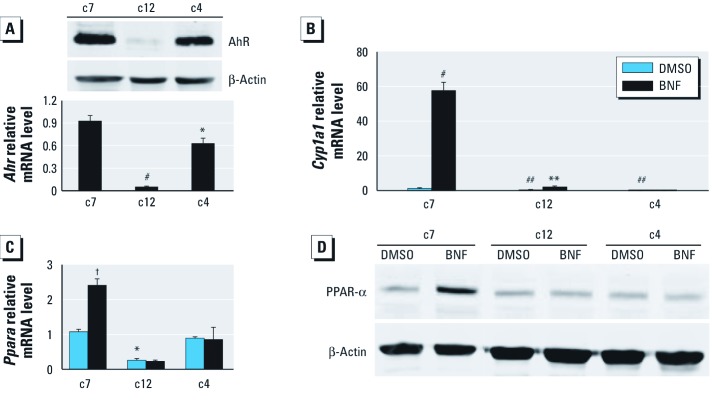
Role of PPAR‑α in *Ahr*-mediated changes in c7, c12, and c4 cells treated with DMSO or 10 µM BNF. (*A*) AhR protein (top) and *Ahr*mRNA (bottom). (*B* and *C*) qPCR analysis of *Cyp1a1* (*B*) and *Ppara *(*C*). (*D*) Immunoblot analysis of PPAR-α protein. *n* = 3 for each cell type and treatment. *p < 0.05 compared with c7 cells. **p < 0.01 compared with DMSO. ^#^*p* < 0.001 compared with c7 cells. ^##^*p* < 0.01 compared with c7 cells. ^†^*p* < 0.05 compared with DMSO.

**Figure 4:**
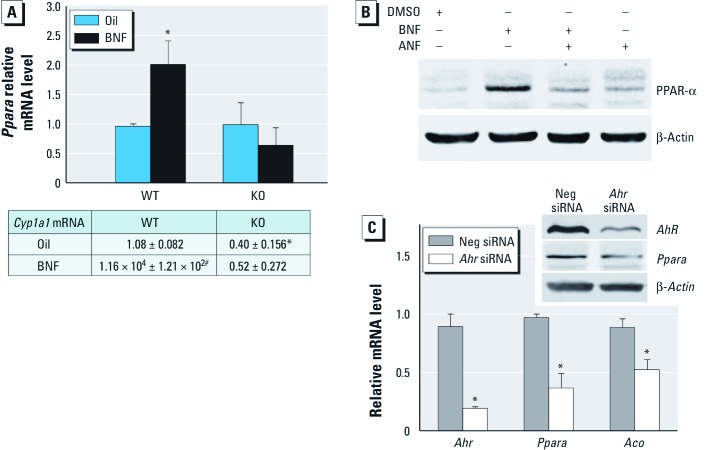
Effect of AhR signaling on PPAR-α expression. (*A*) qPCR analysis of *Ppara* (top) and *Cyp1a1* (bottom) in WT and KO mice (*n* = 4 each) after treatment with oil vehicle or BNF for 24 hr at ZT0. (*B*) Immunoblot showing PPAR-α protein in c7 cells treated with or without DMSO alone or with 10 μM BNF and/or 500 nM ANF. (*C*) qPCR analysis of *Ahr*, *Ppara*, and *Aco* in Ahr-silenced c7 cells (*n* = 3 each) and immunoblot of *Ppara* and *AhR*. *n* = 3 for each cell type and treatment. Data are mean ± SE. Neg, negative. *p < 0.05 compared with WT oil. ^#^*p* < 0.001 compared with WT oil. ^†^*p* < 0.05 compared with Neg siRNA.

*AhR/ARNT signal-pathway–dependent induction of PPAR-*α. Knockout of *Ppara* prevents insulin resistance in mice (Guerre-Millo et al. 2001; Tordjman et al. 2001; Xu et al. 2004). We determined the role of PPAR-α in AhR-mediated changes in glucose metabolism using cell lines (c7, c12, and c4), which have been extensively used to explore AhR/ARNT signaling. c12 cells are c7 derivatives that express extremely low levels of AhR (Figure 3A) (Zhang et al. 1996). The c4 cells are c7 derivatives that lack functional ARNT (Numayama-Tsuruta et al. 1997). As expected, the AhR target gene *Cyp1a1* (cytochrome P450, family 1, subfamily a, polypeptide 1) was significantly reduced in c12 and c4 cells compared with c7 cells after treatment with the AhR agonist BNF (Figure 3B). BNF induced *Ppara* mRNA (Figure 3C) and PPAR-α protein expression (Figure 3D) only in c7 cells (Figure 3C,D). BNF also increased *Ppara* mRNA levels in WT but not in KO mice (Figure 4A). Similar to results in KO mice, basal *Ppara* transcripts were significantly lower in c12 cells than in c7 cells (Figure 3C), and the AhR antagonist ANF blocked BNF-induced PPAR-α expression (Figure 4B). AhR silencing in c7 cells significantly decreased *Ppara* and *Aco* transcription (Figure 4C).

Another AhR ligand, TCDD, also increased *Ppara* mRNA in c7 cells, which returned to baseline by 24 hr [see Supplemental Material, Figure 2a (http://dx.doi.org/10.1289/ehp.1103593)]. In contrast, BNF caused a sustained increase in *Ppara* mRNA (see Supplemental Material, Figure 2a). AhR degradation, which was significantly greater after TCDD treatment (see Supplemental Material, Figure 2b), may explain the difference between agonists. However, both TCDD and BNF induced *Cyp1a1* for the full 24-hr period (see Supplemental Material, Figure 2c). Thus, AhR/ARNT signaling regulates PPAR-α expression.

**Figure 5:**
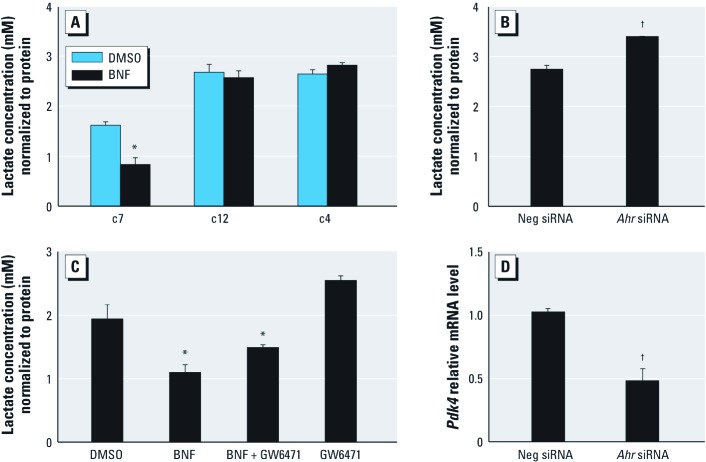
Effect of AhR activation on glycolysis. (*A*) Intracellular lactate concentrations in c7, c12, and c4 cells after treatment with DMSO or 10 μM BNF. (*B*) Lactate levels in *Ahr*-silenced c7 cells. (*C*) Lactate in c7 cells after treatment with DMSO, 10 μM BNF, 10 μM GW6471, or BNF + GW6471. (*D*) qPCR analysis of *Pdk4* mRNA in *Ahr*-silenced c7 cells. Data are mean ± SE; *n* = 3 for each cell type and treatment. Neg, negative. *p < 0.05 compared with DMSO. ^†^*p* < 0.05 compared with Neg siRNA.

*PPAR-*α*–dependent regulation of AhR in glucose metabolism.* Increased PPAR-α in adipocytes increases fatty acid uptake and oxidation and suppresses glucose use (Finck et al. 2005; Ribet et al. 2010). Because PPAR-α levels are regulated by AhR, we examined the effect of AhR activation on glycolysis. BNF (10 μM, 48 hr) reduced intracellular lactate concentration in c7 cells but not in c12 or c4 cells (Figure 5A). *Ahr* silencing significantly increased lactate concentrations in c7 cells, confirming that AhR regulates glycolysis (Figure 5B). To verify the importance of PPAR-α in glycolysis, we used GW6471, a PPAR-α antagonist (Xu et al. 2002). GW6471 did not significantly alter basal levels of intracellular lactate (*p* = 0.0624) but attenuated the BNF-induced decrease in intracellular lactate concentration (Figure 5C). *Pdk4* transcripts were decreased in Ahr-silenced c7 cells (Figure 5D).

**Figure 6:**
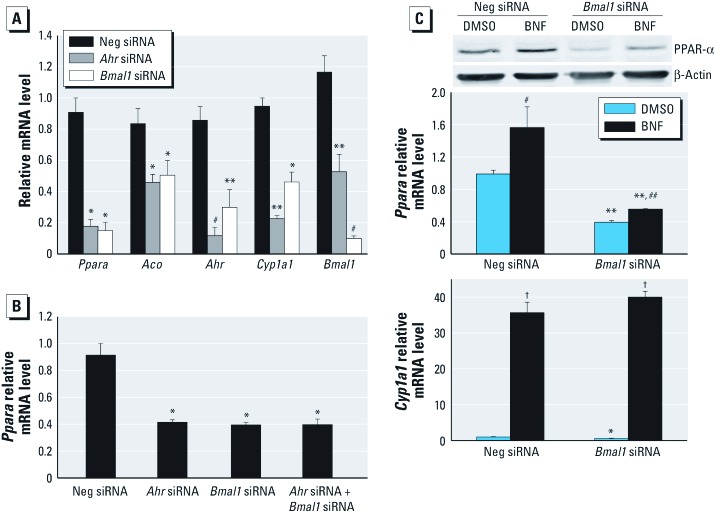
Effect of BMAL1 on AhR-mediated *Ppara *expression. (*A*) qPCR analysis of *Ppara*, *Aco*, *Ahr*, *Cyp1a1*, and *Bmal1* in *Ahr*- or *Bmal1*-silenced c7 cells (*n* = 4 each). (*B*) qPCR analysis of *Ppara* in c7 cells transfected with Neg, *Ahr,* and/or *Bmal1* siRNA (*n* = 3 each). (*C, D*) qPCR analysis and immunoblot of PPAR-α (*C*) and CYP1A1 (*D*) in *Bmal1*-silenced and/or BNF-treated c7 cells (*n* = 5 each). Data are mean ± SE. Neg, negative. *p < 0.05, **p < 0.01, and ^#^*p* < 0.001 compared with Neg siRNA. ^##^*p* < 0.05, ^†^*p *< 0.01, compared with DMSO.

*Involvement of BMAL1 in the regulation of AhR effects on PPAR-*α. CLOCK/BMAL1 regulates PPAR-α expression (Oishi et al. 2005). To confirm that BMAL1 regulates PPAR-α, we silenced *Bmal1* with siRNA. As expected, reduction of BMAL1 decreased PPAR-α and its target gene *Aco* (Figure 6A). Because BMAL1 associates with AhR (Xu et al. 2010), we examined how silencing of each affects PPAR-α expression. Interestingly, *Bmal1* silencing significantly reduced *Ahr* mRNA and suppressed *Cyp1a1* (Figure 6A). *Ahr* silencing also inhibited *Bmal1* mRNA (Figure 6A). Simultaneous silencing of *Bmal1* and *Ahr* was not additive (Figure 6B). Furthermore, *Bmal1* silencing partially blocked PPAR-α induction by BNF (Figure 6C) but did not affect *Cyp1a1* (Figure 6D), showing that BMAL1 is needed for AhR-mediated PPAR-α expression.

Because BMAL1 and AhR both up-regulate PPAR-α, we cotransfected *Ahr* siRNA and *Bmal1* plasmid or *Bmal1* siRNA and *Ahr* plasmid and measured *Ppara* mRNA levels. Overexpression of AhR increased *Cyp1a1* mRNA levels, and *Bmal1* transfection increased *Bmal1* mRNA levels [see Supplemental Material, Figure 3b,c (http://dx.doi.org/10.1289/ehp.1103593)]. However, transfection of *Ahr* and *Bmal1* plasmids did not rescue *Ppara* mRNA down-regulation induced by silencing either *Bmal1* or *Ahr* (see Supplemental Material, Figure 3a).

**Figure 7:**
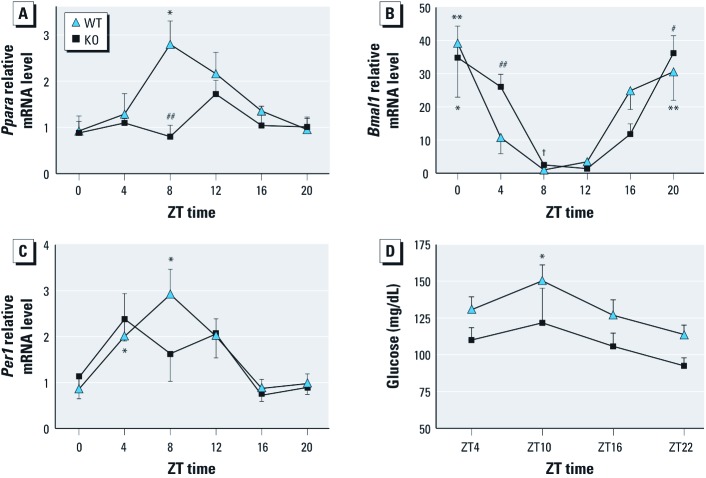
Effect of AhR on (*A*–*C*) circadian rhythm of *Ppara* (*A*), *Bmal1* (*B*), and *Per1* (*C*) expression and on (*D*) rhythm of blood glucose in KO and WT mice. Data are mean ± SE. For *A*–*C*, *n* = 5 KO mice and 4 WT mice; for *D*, *n* = 6 for each. *p < 0.05, **p < 0.01, and ^#^*p* < 0.001 for peak compared with trough. ^##^*p* < 0.05 compared with WT. ^†^*p* < 0.01 compared with WT.

*Altered circadian rhythm in KO mice.* To link AhR to clock and glucose metabolism, we examined rhythms in clock and metabolic genes and blood glucose in KO mice. *Ppara* transcript rhythmicity in WT mice was consistent with results of a previous study (Oishi et al. 2005) but was altered in KO mice (Figure 7A); the amplitude of the *Ppara* rhythm was significantly blunted in KO mice. Rhythms of *Bmal1* and *Per1* mRNA were also slightly altered in KO mice (Figure 7B,C) specifically at ZT4 and ZT8. The rhythm of blood glucose was also attenuated (Figure 7D); glucose in KO mice was almost significantly lower than WT mice at ZT22 (*p* = 0.052).

## Discussion

**Figure 8:**
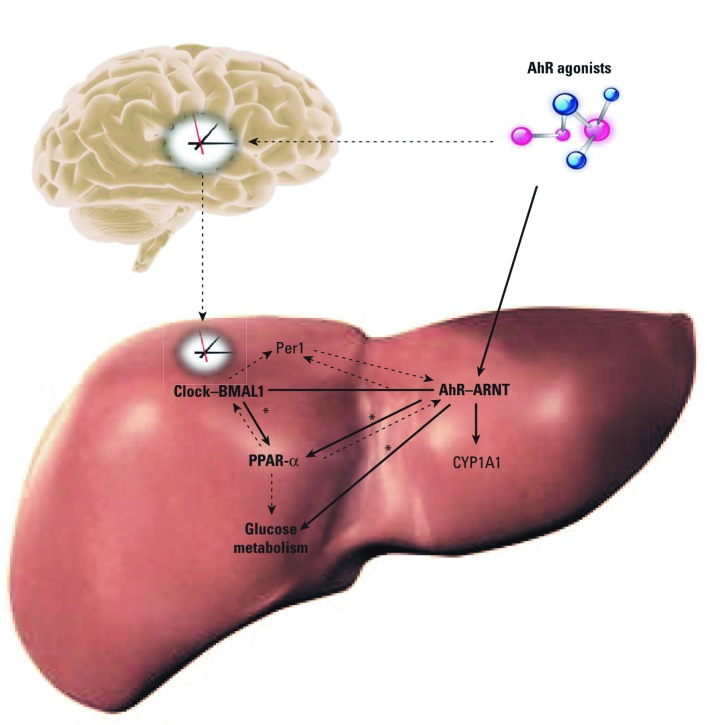
Model of crosstalk among AhR, PPAR-α, and circadian pathways. AhR acts on the liver to regulate PPAR-α and affect circadian rhythms. In turn, PPAR-α influences AhR and BMAL1 levels. PPAR-α levels alter glucose metabolism and insulin sensitivity. *Results from this study.

We examined the interactions of AhR with PPAR-α, glucose metabolism, and circadian rhythm in liver (Figure 8). Liver PPAR-α expression and rhythms are altered after AhR deletion, demonstrating that AhR may be an integral regulator of PPAR-α and may influence liver circadian rhythms (Figure 8, solid arrows). PPAR-α influences both BMAL1 and AhR (Canaple et al. 2006; Villard et al. 2007; see also Figure 6). PPAR-α has been associated with development of insulin resistance (Finck et al. 2005; Koo et al. 2004).

KO mice have enhanced insulin sensitivity and improved glucose tolerance (Figure 1), accompanied by decreased expression of *Ppara* and metabolism genes *Aco*, *Cpt1b*, *G6pase*, and *Pepck* (Figures 2 and 4B). Long-term activation of AhR results in increased peripheral fat mobilization and decreased body fat (Lee et al. 2010). ACO and CPT1b are key enzymes regulating fatty acid β-oxidation. PPAR-α activation increases PEPCK and G6Pase expression, which causes hyperglycemia and insulin resistance (Bernal-Mizrachi et al. 2003). Collectively, these results suggest that KO mice are protected against insulin resistance. Reduced PPAR-α provides a possible mechanism for this protection.

AhR activation up-regulates *Ppara* when AhR signaling is intact (Figure 3C,D). BNF also stimulates *Ppara* expression in WT but not in KO mice (Figure 4A). Moreover, the AhR antagonist ANF blocks the BNF induction of PPAR-α (Figure 4B). Thus, PPAR-α is regulated by AhR/ARNT. Similarly, the AhR ligand 3-methylcholanthrene increases PPAR-α expression in C57Bl/6J mice (Kawano et al. 2010). In contrast, the AhR ligand Sudan III inhibits PPAR-α expression in rats and HepG2 hepatocellular liver carcinoma cells (Shaban et al. 2004). Results of those studies do not necessarily refute our findings because some AhR ligands can activate other signaling pathways (Terzuoli et al. 2010) Transgenic mice with constitutively active AhR have decreased PPAR-α levels (Lee et al. 2010). The constitutively active AhR is missing the minimal ligand-binding domain and binds only to prototypical dioxin response elements. Although AhR binding to alternative promoter elements remains underinvestigated, AhR can interfere with the BMAL1/CLOCK binding to an E-box (Xu et al. 2010). BMAL1/CLOCK also regulates PPAR-α expression through E-box elements (Oishi et al. 2005). Thus, constitutively active AhR may differ from native AhR activation.

TCDD induced *Ppara* mRNA in c7 cells, but *Ppara* returned to basal levels after 24 hr, possibly due to degradation of AhR protein after TCDD treatment [see Supplemental Material, Figure 2a,b (http://dx.doi.org/10.1289/ehp.1103593)]. However, *Cyp1a1* continually increased after BNF or TCDD treatment (see Supplemental Material, Figure 2c), suggesting that mechanisms regulating *Ppara* and *Cyp1a1* after AhR activation are different. PPAR-α activation inhibits glycolysis (Ribet et al. 2010); *Ppara-*null mice exhibit higher rates of glucose metabolism (Knauf et al. 2006; Walker et al. 2007). BNF inhibited glycolysis in c7 cells (Figure 5A); furthermore, the PPAR-α antagonist GW6471 blocked this inhibition (Figure 5C). AhR knockdown elevated lactate concentrations (Figure 5B) and inhibited *Pdk4* mRNA levels (Figure 5D). In contrast *Pdk4* mRNA levels did not change in KO mice (Figure 2C), perhaps because of compensatory mechanisms.

This study confirms that BMAL1 regulates PPAR-α. *Bmal1* silencing decreases c7 *Ppara* and *Aco* (Figure 6A). Surprisingly, *Ahr* mRNA is also decreased after *Bmal1* silencing (Figure 6A). *Cyp1a1* is suppressed, indicating that BMAL1 depletion inhibits AhR (Figure 6A). Conversely, *Bmal1* mRNA significantly decreases in *Ahr*-silenced cells (Figure 6A). Simultaneous silencing of *Ahr* and *Bmal1* does not enhance the inhibitory effect on PPAR-α caused by AhR or BMAL1 knockdown alone (Figure 6B). In *Bmal1*-silenced c7 cells, the induction of PPAR-α by BNF is partially blocked (Figure 6C). Cotransfection of *Ahr* siRNA and *Bmal1* plasmid or *Bmal1* siRNA and *Ahr* plasmid does not rescue *Ppara* down-regulation induced by silencing of *Ahr* or *Bmal1* [see Supplemental Material, Figure 3 (http://dx.doi.org/10.1289/ehp.1103593)]. Collectively, these results provide a possible mechanism for AhR and BMAL1 to regulate PPAR-α. Because PPAR-α regulates BMAL1, AhR, and CYP1A1 (Canaple et al. 2006; Seree et al. 2004; Villard et al. 2007), we speculate that the inhibitory effect of *Bmal1* silencing on AhR and CYP1A1 may be mediated by PPAR-α. Similarly, the reduction in BMAL1 caused by *Ahr* silencing may depend on PPAR-α. AhR and PPAR-α may provide positive feedback to the liver circadian clock.

AhR and ARNT exhibit strong rhythmicity in liver (Mukai et al. 2008), but the underlying mechanism remains unclear. Disruption of Per1 and Per2 alters AhR (Qu et al. 2007). AhR activation changes the expression of liver Per1 and BMAL1. Although in KO mice *Bmal1* and *Per1* rhythms are only modestly altered, the diurnal variations in blood glucose and *Ppara* are significantly changed (Figure 7). Although fasting glucose levels did not change, KO mice showed an enhanced glucose and insulin sensitivity compared with WT mice (Figure 1A,C). Thus, AhR may regulate circadian clock output in peripheral clock systems such as the liver.

## Conclusions

Glucose metabolism in KO mice is reminiscent of that in PPAR-α–deficient mice; AhR clearly regulates PPAR-α. Future studies will explore how AhR and BMAL1 regulate PPAR-α expression in other peripheral tissues, such as pancreas, muscle, and adipose. It will also be important to develop *in vivo* toxicant exposure models to mimic development of diabetes caused by chronic environmental pollutant exposure in humans.

The epidemic of obesity and diabetes is widely recognized as an emerging public health problem of enormous magnitude. AhR activation, although much less widely acknowledged, is also epidemiologically associated with diabetes (Bock 1994). These pollutants are ubiquitous in modern life, and their effects profoundly influence human health. Understanding the mechanisms involved in AhR-induced diabetes has the potential to revolutionize our understanding of type 2 diabetes.

## Supplemental Material

(393 KB) PDF
