# Supplementary material for: Aryl Hydrocarbon Receptor Deficiency Enhances Insulin Sensitivity and Reduces PPAR-α Pathway Activity in Mice
Source: Environ Health Perspect. 2011 Aug 17;119(12):1739–44. doi: 10.1289/ehp.1103593 (PMC3261983; doi:10.1289/ehp.1103593)
Supplement: (393 KB) PDF [file ehp.1103593.s001.pdf]

Supplemental Material for:

Aryl Hydrocarbon Receptor Deficiency Enhances Insulin Sensitivity and Reduces PPAR-α Pathway Activity in Mice

Chun Wang[1,3*], Can-Xin Xu[1,3*], Stacey L. Krager[2], Kathleen M. Bottum[2], Duan-Fang Liao [4] & Shelley A. Tischkau[1]

*These authors contributed equally.

[1] Department of Pharmacology, Southern Illinois University School of Medicine, Springfield, Illinois, USA.

[2] Department of Internal Medicine, Southern Illinois University School of Medicine, Springfield, Illinois, USA.

[3]Institute of Pharmacy and Pharmacology, College of Pharmacy and Life Science, University of South China, Hengyang, Hunan, China.

[4] Division of Stem Cell Regulation and Application, State Key Laboratory of Chinese Medicine Powder and Medicine Innovation in Hunan(incubation), Hunan University of Chinese Medicine, Changsha, Hunan, China.

Corresponding Author: Shelley A. Tischkau, 801 N Rutledge Rm3354, PO Box 9629, Springfield, Illinois 62974-9629, USA. 217-545-6524, Fax: 217-545-0145, stischkau@siumed.edu.

Supplemental Material Table of Contents

Item **Page Number**

Supplemental Material, Table……………………………………….…………………….2

Supplemental Material, Figure 1…………………………………….……………….3

Supplemental Material, Figure 2……………………………………………………..4

Supplemental Material, Figure 3……………………………………………………..5

References………………………………………………………………………………6

Supplemental Material, Table

Gene	Direction	Sequence	Source
Ahr siRNA	Sense<br>Antisense	GCAGAUGCCUUGGUCUUCUAUGCUU<br>AAGCAUAGAAGACCAAGGCAUCUGC	Invitrogen
Bmal1 siRNA	Sense<br>Antisense	UGGACGAAGACAAUGAGCCAGACAA<br>UUGUCUGGCUCAUUGUCUUCGUCCA	Invitrogen
Pepck primers	Forward<br>Reverse	5'-CCACAGCTGCTGCAGAACA-3'<br>5'-GAAGGGTCGCATGGCAAA-3'	Stone et al. 2004
G6pase primers	Forward<br>Reverse	5'-TGCAAGGGAGAACTCAGCAA-3'<br>5'-GGACCAAGGAAGCCACAATG-3'	Bernal-Mizrachi et al. 2003
Aco primers	Forward<br>Reverse	5'-ATTCTCACAGCAGTGGGATTCC-3<br>5'-CTGCAGCATCATAACAGTGTTCTC-3'	Tordjman et al. 2001
Ppar-α primers	Forward<br>Reverse	Proprietary<br>Proprietary	Qiagen
Pdk4 Primers	Forward<br>Reverse	Proprietary<br>Proprietary	Qiagen
Cpt1b primers	Forward<br>Reverse	Proprietary<br>Proprietary	Qiagen

Supplemental Material, Table. Sequences for antisense and primers.

Sequences are given for the antisense and primers indicated, along with their source. Qiagen sequences are proprietary.

Supplemental Material, Figure 1

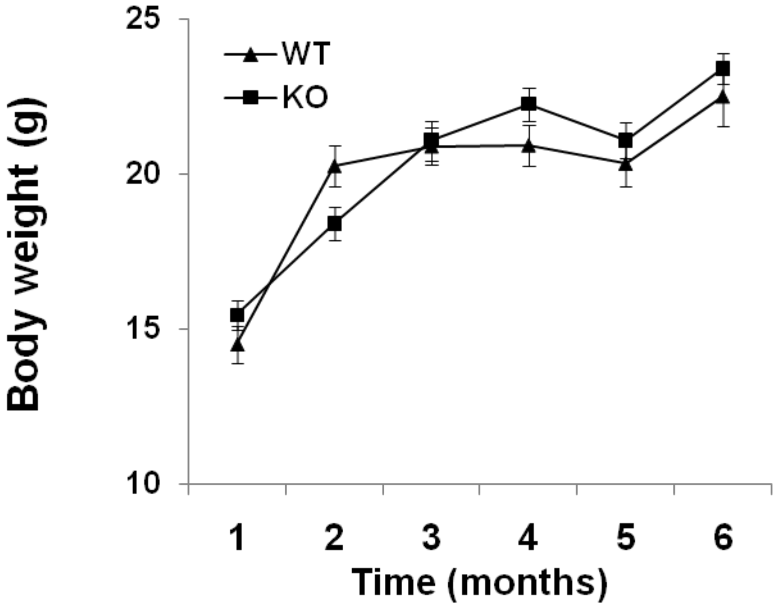

Supplemental Material, Figure1. **Average body weights of WT and AhRKO mice.**

Mice were weighed at 1, 2, 3, 4, 5 and 6 months of age (n=4 each). **KO**: AhR deficient animal (knockout).

Supplemental Material, Figure 2

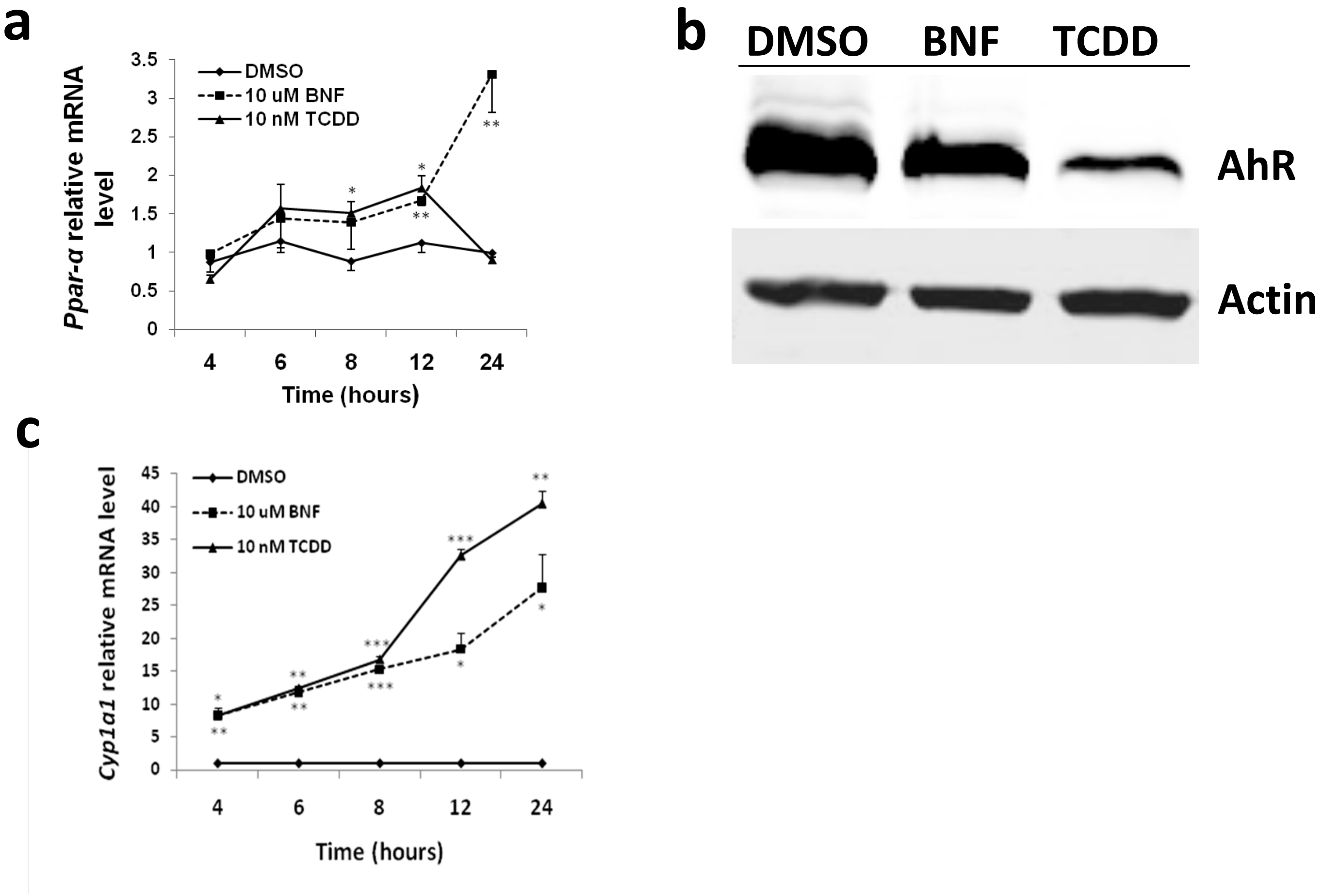

Supplemental Material, Figure 2. Effect comparison of TCDD and BNF on PPAR-α expression.

a. Time courses of *Ppar-α* mRNA levels in 10 nM TCDD or 10 μM BNF treated c7 cells. **b**. AhR protein expression in c7 cells treated with 10 nM TCDD or 10 μM BNF for 24 hr. **c**. *Cyp1A1* mRNA levels in cells described in **a**. Data are presented as mean ± SEM of three replicates. *, $P < 0.05$, **, $P < 0.01$ and ***, $P < 0.001$ vs. DMSO control at different time points.

Supplemental Material, Figure 3

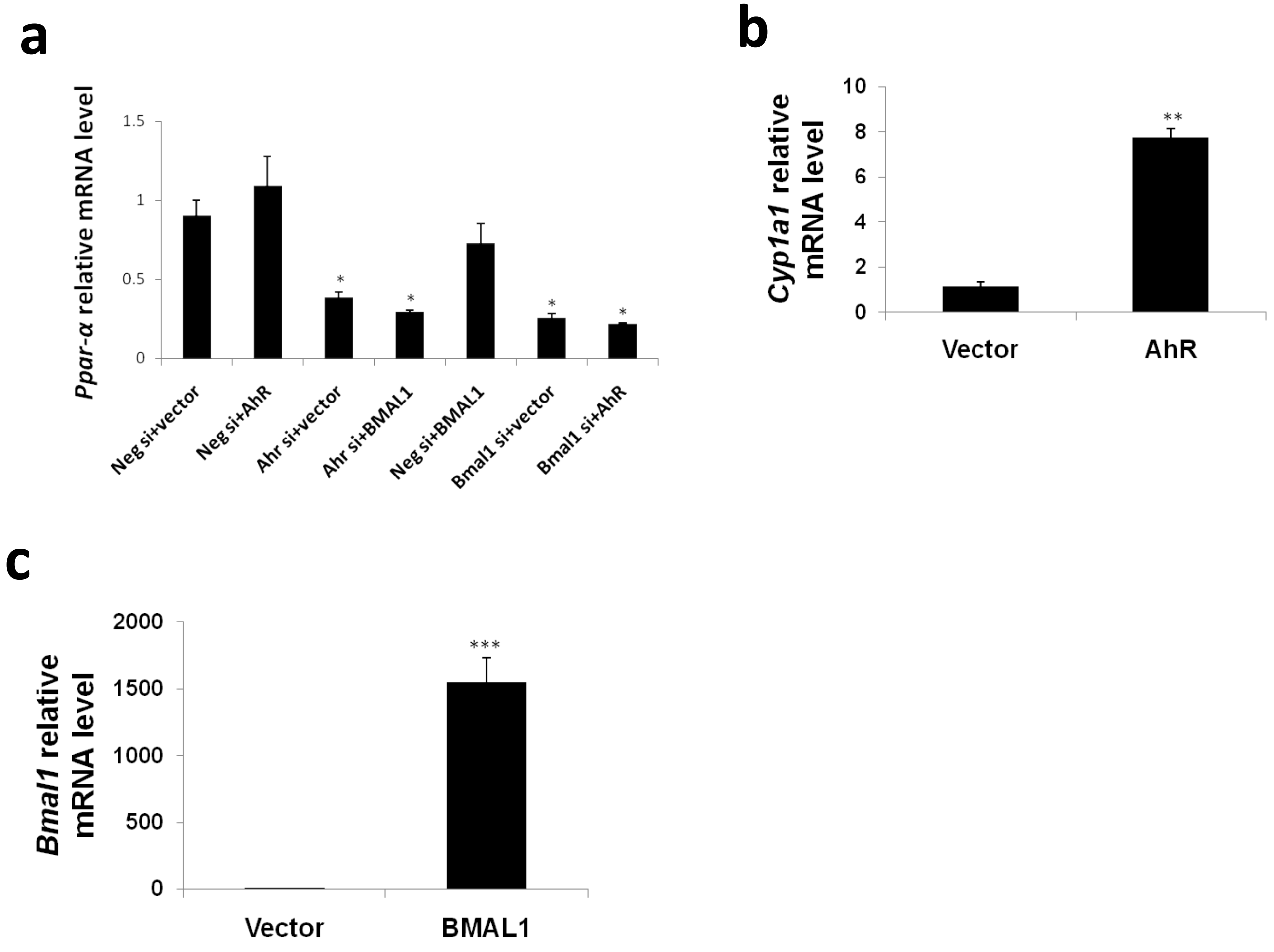

Supplemental Material, Figure 3. Effect of co-transfection of AhR/BMAL1 plasmid and BMAL1/AhR siRNA on PPAR-α expression.

a. qPCR analysis of *Ppar-α* in *Ahr*/*Bmal1* plasmid and *Bmal1*/*Ahr* siRNA co-transfected c7 cells. **b**. qPCR analysis of *Cyp1A1* in c7 cells transfected with *Ahr* plasmid. **c**. qPCR analysis of *Bmal1* in c7 cells transfected with *Bmal1* plasmid. Data are presented as mean ± SEM of three replicates. *, $P < 0.05$, **, $P < 0.01$ and ***, $P < 0.001$ vs. Neg si and/or vector control. **AhR si:** AhR siRNA; **BMAL1 si**: BMAL1 siRNA ; **Neg si:** negative siRNA; **AhR:** *Ahr* plasmid; **BMAL:** *Bmal1* plasmid.

References

Bernal-Mizrachi C, Weng S, Feng C, Finck BN, Knutsen RH, Leone TC, et al. 2003. Dexamethasone induction of hypertension and diabetes is PPAR-alpha dependent in LDL receptor-null mice. Nat Med 9(8):1069-1075.

Stone SJ, Myers HM, Watkins SM, Brown BE, Feingold KR, Elias PM, et al. 2004. Lipopenia and skin barrier abnormalities in DGAT2-deficient mice. J Biol Chem 279(12):11767-11776.

Tordjman K, Bernal-Mizrachi C, Zemany L, Weng S, Feng C, Zhang F, et al. 2001. PPARalpha deficiency reduces insulin resistance and atherosclerosis in apoE-null mice. J Clin Invest 107(8):1025-1034.
